# Machine Learning Models Leveraging Smartphone-Based Patient Mobility Data Can Accurately Predict Functional Outcomes After Spine Surgery

**DOI:** 10.3390/jcm13216515

**Published:** 2024-10-30

**Authors:** Hasan S. Ahmad, Daksh Chauhan, Mert Marcel Dagli, Ryan W. Turlip, Malek Bashti, Ali Hamade, Patrick T. Wang, Yohannes Ghenbot, Andrew I. Yang, Gregory W. Basil, William C. Welch, Jang Won Yoon

**Affiliations:** 1 Department of Neurosurgery, Perelman School of Medicine, University of Pennsylvania, Philadelphia, PA 19104, USA; 2 Department of Neurosurgery, Miller School of Medicine, University of Miami, Miami, FL 33136, USA; 3 Department of Neurosurgery, Barrow Neurological Institute, Phoenix, AZ 85013, USA

**Keywords:** lumbar decompression, lumbar fusion, lumbar spine, machine learning, patient reported outcome measures, smartphone-based accelerometry

## Abstract

**Objective:** The development of adjacent segment disease or the progression of spondylosis following the surgical treatment of spinal stenosis and spondylolisthesis is well documented and can lead to subsequent functional decline after a successful index surgery. The early detection of negative inflection points during patients’ functional recovery can improve timely intervention. In this study, we developed machine learning (ML) models to predict the occurrence of post-operative decline in patient mobility. **Methods:** Patients receiving spine surgery for degenerative spinal stenosis or spondylolisthesis were retroactively consented and enrolled. Activity data (steps-per-day) previously recorded across a 4-year peri-operative were collected alongside relevant clinical and demographic variables. Logistic regression (LR), random forest (RF), and extreme gradient boosting (XGBoost) ML models were constructed and trained on 80% of the dataset and validated using the remaining 20%. The study’s primary endpoint was the models’ ability to predict post-operative decline in patient mobility. **Results:** A total of 75 patients were included. Following training, RF and XGBoost models achieved accuracy values of 86.7% (sensitivity 80%, specificity 90%) and 80% (sensitivity 60%, specificity 90%), respectively, in predicting post-operative functional decline. The LR model was the least effective with an accuracy of 73.3% (sensitivity 50%, specificity 88.8%). Receiver operating characteristic curves showed an area under the curve of 0.80 for RF, 0.70 for XGBoost, and 0.69 for LR. **Conclusions:** ML models trained on activity data collected from smartphones successfully forecast functional decline in post-operative activity following spine surgery. These results lay the groundwork for the future integration of ML into the surgeon’s toolbox for prognostication and surgical planning.

## 1. Introduction

The efficacy of spine surgical intervention in the management of conditions such as degenerative spinal stenosis and spondylolisthesis has been well established in neurosurgical literature [1,2,3]. The current “gold standard” of outcome measures across this body of work is patient-reported outcome measures (PROMs). These commonly employed surveys and questionnaires, such as the Oswestry Disability Index (ODI) [4] and EuroQOL-5D (EQ-5D) [5], are established as reliable and valid methods of assessing a patient’s personal perception of pain, mobility, and ability to complete activities of daily living at a discrete point in time [6]. Despite the success and relative ubiquity of these outcome measures, their subjective and discrete nature render them sub-optimal for truly quantitative assessments and comparisons of surgical efficacy [7,8].

Growing awareness of the current inadequacy in capturing the temporal and graded nature of post-operative recovery has given rise to an emerging paradigm of activity- and mobility-based metrics to measure and report the functional improvements of patients after surgery [9,10,11]. This body of work uses a range of monitoring devices, from externally worn accelerometers to patient smartphones, to quantify the daily physical activity of individual patients across the peri-operative window. By providing a vast amount of granular, unbiased, and seamlessly collected data, these objective metrics supplement traditional PROMs by allowing surgeons to understand not just how the patient is feeling but exactly how often and the degree to which the patient is mobilizing.

Precise and data-driven outcome methodologies are informative across the breadth of spine surgical practice, from pre-operative prognostication based on a more accurate understanding of a patient’s baseline functional status [12] to post-operative monitoring of patient progression and surveillance for symptom recurrence, and even comparing the relative efficacy of new and existing surgical techniques. Despite this existing work in the pre-operative and immediate post-operative periods, the possibility of using pre-operative patient activity data to predict post-operative success and improve prognostication remains to be fully explored. Unlike comparative studies, the accuracy and clinical usefulness of predictive models is much more dependent on the volume and variety of data provided, which quickly saturates and surpasses the computational ability of humans and basic mathematical techniques [13]. To fully capitalize on and extract insights from the datasets generated by functional outcome metrics, machine learning (ML)—a subset of artificial intelligence comprised of algorithms that learn and self-adjust to optimize the mapping of input data to an output prediction [14]—can be used. In this study, we utilize high-fidelity patient activity data to predict the likelihood of sustained improvement in physical mobility after surgery through multimodal ML models trained and validated on both clinical and functional patient variables.

## 2. Materials and Methods

### 2.1. Patient Selection and Data Source

Patients who received spinal surgery between 1 January 2014, and 1 July 2020 at two urban academic centers were eligible for enrollment in this retrospective study. Inclusion criteria comprised patients with clinical symptoms and radiographic findings that merited elective spine surgery as well as ownership and the consistent usage of an Apple iPhone (Apple Inc., Cupertino, CA, USA). Patients meeting these criteria were contacted and consented over the telephone and in person. Consenting patients were instructed to download QS Access (Quantified Self Labs, San Francisco, CA, USA), which is a mobile application that collates and securely exports Apple Health activity data. The primary variable extracted and analyzed in this study was patients’ daily step count. Additional demographic and clinical variables were also collected from the electronic medical record (EHR). This study was approved by our Institutional Review Board (IRB #843229). This study’s design and reporting were supported by guidelines from Strengthening the Reporting of Observational Studies in Epidemiology (STROBE) and Transparent Reporting of a Multivariable Prediction Models for Individual Prognosis or Diagnosis (TRIPOD) [15,16].

### 2.2. Variables

**Figure 1:**
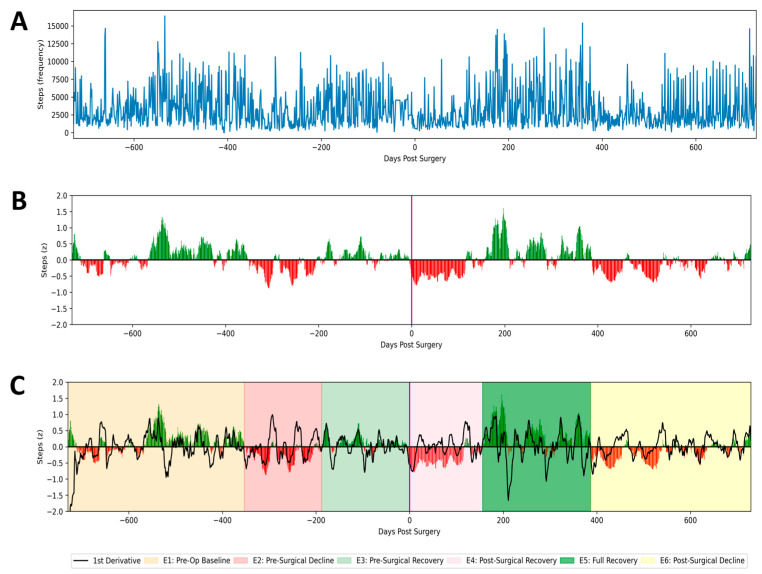
An example patient’s activity (i.e., steps taken per day) is collected across a 4-year peri-operative window (**A**), smoothed and normalized to each individual’s pre-operative baseline (**B**), and subsequently segregated into distinct temporal epochs based on the activity magnitude and rate of change (**C**).

Baseline characteristics were extracted from EHRs, including age (years), sex, body mass index (BMI), and operated levels. Using patient activity data, a time series of steps-per-day was constructed across a 4-year peri-operative window, spanning 2 years prior through 2 years following a patient’s date of surgery (Figure 1A). To account for inter-patient variability in absolute activity levels and to permit subgroup analysis, patient step counts were normalized with respect to the first year of patient data, representing each patient’s true baseline activity level. These data were then smoothened using a 14-day sliding window to minimize noise and amplify true signals (Figure 1B).

A previously developed and validated algorithm [11] was applied to each patient time series to classify physical activity into six distinct temporal epochs (Figure 1C):

(1) Pre-operative baseline, as described above;
(2) Acute pre-operative decline;
(3) Periods of spontaneous recovery of activity;
(4) Acute post-operative recovery;
(5) Fully recovered state with post-operative activity levels commensurate to or exceeding pre-operative baseline; and
(6) Secondary decline in activity following prior compete post-operative recovery.

Following this segmentation, the total cumulative days patients remained in each epoch, the rate of activity change within each epoch (i.e., the 1st derivative), and the change in the rate of activity itself (i.e., the 2nd derivative) were calculated.

The primary objective of this study was to predict the occurrence of secondary post-operative functional decline, which was defined by a 0.25 standard deviation or greater decrease in a patient’s mean steps-per-day following a complete surgical recovery. Clinically, this would manifest in patients as reduced ambulatory capacity due to pain or weakness.

### 2.3. Statistics

Three distinct ML algorithms were constructed and implemented: logistic regression (LR), random forest (RF), and extreme gradient boosting (XGBoost) models. LR is a statistical technique that estimates the likelihood that a dichotomous categorical outcome variable occurs given certain characteristics of the predictor set by plotting the natural logarithm of the odds of the dependent variable as a linear combination of the input features [17]. The simplicity of LR renders it computationally efficient, though it tends to overfit predictions based on the feature set. In contrast, RF and XGBoost are truer ML algorithms that use ensemble classification architecture, whereby multiple classifiers work in unison to create a set of decision trees [18,19]. RF uses predictive voting to aggregate trees, whereas XGBoost sequentially optimizes subsequent decision trees based on loss function minimization. Both methods introduce feature randomness and regularization to reduce overfitting and are better suited to capture non-linear relationships.

The input variables included the activity parameters above as well as patient BMI and age at surgery. The patient dataset was split into a training set and validation set using a 4:1 ratio. Model efficacy was defined as the accuracy of predicting the occurrence of a secondary decline in activity following prior sustained recovery. Differences in performance were compared using each model’s accuracy, sensitivity, specificity, positive predictive value, negative predictive value, and area under the curve (AUC) of the receiver operating characteristic. All analyses were conducted utilizing R 4.3 (R Foundation, Vienna, Austria) and Python 3.7 (Python Foundation, Wilmington, DE, USA).

## 3. Results

### 3.1. Baseline Demographics

A total of 75 patients were included in this study with surgeries including lumbar fusion (LF; 34), lumbar decompression (LD; 28), cervical fusion (7), cervical decompression (2), cervical total disc arthroplasty (1), and lumbar kyphoplasty (3). The cohort was 42% male and 58% female with the average age at surgery being 61.3 years old and average BMI being 29.4 (Table 1). Across patients, 52.2% received a single-level surgery, 17.4% received a two-level surgery, and 30.4% received a three-level or greater surgery. In aggregate, the step counts for all patients consisted of over 109,000 distinct datapoints.

Across all patients, there were no intra-operative complications. One (1.3%) patient required re-operation for recurrent disc herniation.

### 3.2. Activity Classification

Pre- and post-operative patient activity, derived from mobility data stored on patient smartphones, was classified as detailed in the methodology above. Patients spent an average of 250.0 days in a period of acute pre-operative deterioration of physical mobility compared to their baseline (Table 2). This did not differ between the type of surgery received (*p* = 0.685). Across all patients, 82.67% regained mobility commensurate to or above the pre-operative baseline. Patients who successfully recovered spent an average of 217.0 days regaining functional status. There was no statistically significant difference in normalized activity levels between surgery type across the acute pre-operative decline, post-operative recovery, and fully recovered states (Table 2).

Additionally, 34.7% of patients experienced a post-operative secondary decline in physical activity after previously regaining full physical mobility (15 LF, 11 LD, and 4 other cervical surgeries). The average length of the post-operative recovery and fully recovered periods before secondary decline were 119.7 days and 201.5 days, respectively. LF patients experienced a significantly greater decrease in mobility during these periods of secondary decline compared to LD patients (paired t-test yields *p* = 0.0026).

### 3.3. Model Accuracy

The RF model was the most successful predictive model (86.7% accuracy, 80.0% sensitivity, 90.0% specificity) with XGBoost performing similarly (80.0% accuracy, 60.0% sensitivity, 90.0% specificity). The LR model was least successful (73.3% accuracy, 50.0% sensitivity, 88.8% specificity). All models had greater specificity than sensitivity, indicating greater prediction accuracy for cases where the patient does not experience a secondary post-operative decline (Table 3).

**Figure 2:**
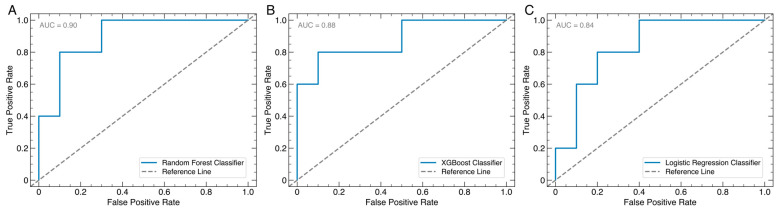
Area under the receiver operating characteristic curve for random forest (**A**), extreme gradient boosting (XGBoost; (**B**)), and logistic regression (**C**) models.

The model performance gauged by the receiver operating characteristic (ROC) curve yielded total area under the curve (AUC) values of 0.90 for RF (Figure 2A), 0.88 for XGBoost (Figure 2B), and 0.84 for LR (Figure 2C), showing that RF exhibited the greatest measure of separability.

### 3.4. Feature Contribution to Decision Tree Models

**Figure 3:**
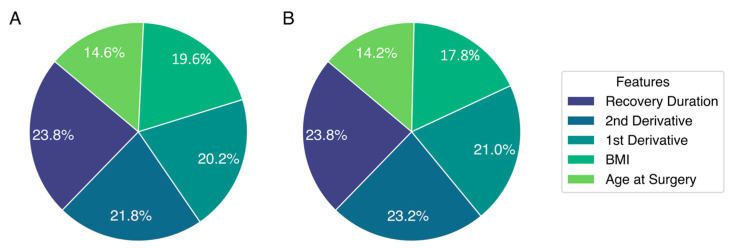
Feature importance for random forest (**A**) and extreme gradient boosting (XGBoost; (**B**)) models, the two decision tree algorithms implemented.

The feature importance for both RF (Figure 3A) and XGBoost (Figure 3B), the two decision tree models used in this analysis, was extracted. For both models, the duration of the immediate post-operative recovery period, when a patient is regaining their baseline physical functionality, contributed toward approximately one quarter of the total prediction. Subsequent analysis showed that a greater post-operative recovery length was correlated with a lesser probability of subsequent functional decline (r2 = −0.349). Other important features for both tree models included age, BMI, and the 1st and 2nd derivatives of the activity time series.

## 4. Discussion

We present a data-driven method to predict post-operative decline in physical mobility in patients undergoing spine surgery. Our three machine learning algorithms utilized clinical variables in conjunction with pre- and post-operative activity data as measured by patient smartphones to determine with a high degree of accuracy whether patients would sustain their post-operative functional improvements or experience a subsequent mobility decline. Random forest modeling was most accurate in this regard (AUC = 0.80, 86.7% accuracy, 80.0% sensitivity, 90.0% specificity) and relied greatly on patients’ length of post-operative recovery, the rate of increasing activity during the recovery period (1st derivative), BMI, and age. Objective activity monitoring also revealed that amongst patients who do not sustain post-operative improvements in mobility, lumbar fusion patients experience greater declines in physical activity compared to lumbar decompression patients (*p* = 0.003). This work illustrates the efficacy of using ML to unlock the full potential of objective outcome metrics in spine surgery and paves the way for future studies with more generalizable results and informative clinical insights.

Traditional methods of quantifying post-operative recovery in spine surgery, such as the ODI4 and EQ-5D [5], all rely on self-reported static questionnaires, which is a methodology that has remained largely unchanged since the inception of PROMs in 1978 [20]. Importantly, these surveys offer a way to systematically collect a patient’s perspective of their disease process, which is crucial in establishing the substrate for shared decision making [21]. However, this strength of PROMs is also its greatest drawback; subjectivity in the collection process of these surveys introduces possible recall and response bias, such as by relying on patients’ historical recollection and recent symptomatology to characterize long-standing and chronic disease processes [8]. Investigations into similar surveys such as the Short-Form Six-Dimension (SF-6D) and Quality of Well Being scale (QWB) have shown that the variability between repeated survey administrations approaches established minimum clinically important differences (MCIDs), showing that clinical decision making based on these and similar instruments may not always be reliable. Additionally, the discrete nature of PROM administrations at specific pre- and post-operative clinic visits provides a very limited window into the true status of a patient’s functional health and well-being.

Objective, mobility-based outcome metrics attempt to address these coverage gaps. Activity data from highly fidelitous sources such as externally worn accelerometers or patient smartphones have been shown to be highly concordant with a patient’s true level of mobility [22]. Using objectively recorded data rather than relying on subjective reporting eliminates potential inaccuracies and increases the validity, reliability, and responsiveness of these novel outcome measures. Additionally, passive data collection allows for finer temporal resolution on the scale of hours and days compared to the weeks and months between PROMs in their current form, imparting a more detailed depiction of patients’ baseline health, disease progression, and post-operative recovery. Early investigations of objective outcome metrics have proven the clinical benefit of this granularity, illustrating that activity data can be used to characterize not only the degree to which patients regain mobility after spine surgery but also the specific pathologic process affecting their spine [11,12].

The next step in the progression and development of these outcome metrics is to work backwards and utilize patient activity data to predict which patients are likely to experience the greatest clinical benefit from spine surgical intervention. Answering this question has tremendous clinical value and paves the way for surgeons to use validated, data-driven methods to inform patient selection and surgical prognostication. As we illustrate in this study, the volume of smartphone-derived data, and not just its objectivity, is where the true advantage of activity-based outcomes lies. Fully unlocking this potential, then, is only feasible using techniques designed to process and harvest insights from these large datasets, namely machine learning. ML has quickly gained prominence in spine surgery research in lieu of traditional statistical techniques, as these tools can extract relationships underlying the inherent complexity of clinical data that are otherwise invisible to the human eye [23].

Amongst our ML models, decision tree classifiers (i.e., random forest and XGBoost) yielded greater predictive performance than logistic regression, which was likely due to their superior processing and iterative understanding of non-linear relationships among the input features. One of the strongest variables contributing to the accuracy of these classifiers was the duration of post-operative recovery with longer recovery periods before reaching and/or exceeding baseline mobility status associated with sustained functional recovery. While this association is not necessarily causal, it raises additional questions for further investigation such as whether certain behavior patterns of patients with faster post-operative recoveries lead to a greater likelihood of subsequent decline, if there is a crucial window within the immediate post-operative period that influences downstream recovery, and whether specific clinician follow-up in the immediate post-operative period can have a disproportionately beneficial impact on patients. Additionally, further investigation is needed to understand why post-operative declines were more severe in lumbar fusion compared to other surgeries; this likely stems from the relatively higher pre-operative disease burden and more invasive operative requirements to decompress and stabilize the spine. While the results of this initial study do merit follow-up, these and subsequent results should be interpreted with caution to avoid over- or under-generalizing the results during clinical decision making. Although ML can enhance prediction, models are inherently imperfect; over-reliance on them could dissuade surgeons from offering, or patients from accepting, otherwise beneficial surgical intervention.

To our knowledge, this is the first study implementing ML using objective, mobility-based data in spine surgery. Across the 29 spine surgery outcome prediction tools available in contemporary literature, all prior studies utilized solely clinical variables such as age, symptom duration, length of stay, and hospital- or surgery-associated complications; Single-institution studies had sample sizes ranging from 27 to 1053 with the total comprised datapoints orders of magnitude less than the dataset used herein [24,25]. Because the relative benefit of ML over traditional statistical measures in the prediction and optimization of signal-to-noise ratios is most dependent on the total number of data input, we believe our study uniquely adds to the current landscape of spine surgery and outcomes prediction [26].

This study must be interpreted within the context of its limitations. Although our models were trained and validated on a very large number of datapoints, the limited patient population and feature set limits broad generalizability. Future studies should primarily aim to include larger numbers of patients from multiple, geographically and demographically diverse institutions, such as multi-institutional datasets and national and international registries. Additionally, while patients receiving different surgeries were grouped together for this initial analysis, subsequent investigations with larger patient cohorts should aim for subgroup analysis within surgical types and techniques so as to better identify and isolate activity trends within specific patient populations. Input features into these predictive models should also be expanded to encompass a greater breadth of variables that could influence overall patient outcomes, including radiographic parameters of the disease process, medical co-morbidities, and cognitive and psychosocial functioning. Self-reported pain and physical mobility, as collected through PROMs, are also important to include in subsequent studies not only to assess the potential predictive overlap between subjective and objective measures of patient mobility but also to validate our initial results with existing measures. Other limitations inherent to retrospective data analysis are also present, such as confounding due to unseen variables or the clustering of patients in the study. Lastly, study enrollment was voluntary, and patients opting to provide their activity data may have a positively skewed relationship between activity and functional outcome, whether due to age, level of smartphone usage, or other factors.

## 5. Conclusions

We demonstrate the ability of machine learning classifiers trained on activity data from patients’ smartphones to accurately predict post-operative functional recovery after spine surgery. This is the first such study to utilize ML with objective outcomes data, and it provides a compelling substrate for future iterations of predictive models that leverage the granularity and temporal specificity of such data. Further training of ML models on larger, multi-institutional datasets and national registries is needed to build more generalizable models.

## Figures and Tables

**Table 1:** Baseline characteristics.

Variable	Lumbar Fusion (N = 34)	Lumbar Decompression (N = 28)	Other (N = 13)
**Sex**			
Male	13 (38.2%)	10 (35.7%)	8 (61.5%)
Female	21 (61.8%)	18 (64.3%)	5 (38.5%)
**Age**			
<50 years	4 (14.3%)	6 (21.4%)	3 (23.1%)
51–60 years	5 (17.9%)	4 (14.3%)	5 (38.5%)
61–70 years	9 (32.1%)	11 (39.3%)	4 (30.8%)
71–80 years	8 (28.6%)	7 (25%)	1 (7.7%)
80+ years	2 (7.1%)	0 (0%)	0 (0%)
**BMI (kg/m^2^)**			
<20	1 (3.6%)	1 (3.7%)	0 (0%)
20–25	9 (32.1%)	9 (33.3%)	3 (23.1%)
25–30	10 (35.7%)	7 (25.9%)	3 (23.1%)
30–35	7 (25%)	4 (14.8%)	6 (46.2%)
35–40	1 (3.6%)	2 (7.4%)	1 (7.7%)
40+	0 (0%)	4 (14.8%)	0 (0%)
**Operated Levels**			
1	17 (60.7%)	15 (53.6%)	4 (40%)
2	8 (28.6%)	2 (7.1%)	2 (20%)
3	3 (10.7%)	10 (35.7%)	3 (30%)
>3	0 (0%)	1 (3.6%)	1 (10%)

Baseline demographics for all included patients. ‘Other’ surgery category includes cervical fusion (7), cervical decompression (2), cervical total disc arthroplasty (1), and lumbar kyphoplasty (3).

**Table 2:** Durations of patient mobility categorizations.

Patient Mobility Categories	Lumbar Fusion (Days ± SE)	Lumbar Decompression (Days ± SE)	Other (Days ± SE)	*p*
Acute Pre-Operative Decline	219.9 (±39)	208.5 (±35)	274.6 (±73)	0.685
Pre-Operative Spontaneous Recovery	112.9 (±26)	189.4 (±45)	137.1 (±45)	0.321
Immediate Post-Operative Recovery	172.3 (±34)	220.0 (±40)	169.3 (±71)	0.635
Fully Recovered	264.3 (±42)	178.8 (±34)	232.9 (±63)	0.332
Post-Operative Secondary Decline	233.0 (±52)	267.5 (±46)	303.5 (±126)	0.904

Patient activity was categorized into distinct epochs, including acute pre-operative decline, periods of spontaneous recovery of activity, acute post-operative recovery, fully recovered state with post-operative activity levels commensurate to or exceeding pre-operative baseline, and secondary decline in activity following prior complete post-operative recovery. Comparisons were tested with an analysis of variance test for statistical significance with an α value of 0.05.

**Table 3:** Model performance metrics.

	Random Forest	XGBoost	Logistic Regression
Accuracy	86.7%	86.7%	86.7%
Sensitivity	80%	80%	80%
Specificity	90%	90%	90%
AUROC	0.90	0.88	0.84
Positive Predictive Value	90%	90%	80%
Negative Predictive Value	90%	90%	90%

Performance metrics for the 3 machine learning models implemented. XGBoost = extreme gradient boosting. AUROC = area under recover operating characteristic.

## Data Availability

The data presented in this study are available on request from the corresponding author due to (specify the reason for the restriction).

## References

[1] Ghogawala Z., Dziura J., Butler W.E., Dai F., Terrin N., Magge S.N., Coumans J.-V.C., Harrington J.F., Amin-Hanjani S., Schwartz J.S. (2016). Laminectomy plus fusion versus laminectomy alone for lumbar spondylolisthesis. N. Engl. J. Med..

[2] Weinstein J.N., Lurie J.D., Tosteson T.D., Hanscom B., Tosteson A.N., Blood E.A., Birkmeyer N.J., Hilibrand A.S., Herkowitz H., Cammisa F.P. (2007). Surgical versus nonsurgical treatment for lumbar degenerative spondylolisthesis. N. Engl. J. Med..

[3] Malmivaara A., Slätis P., Heliövaara M., Sainio P., Kinnunen H., Kankare J., Dalin-Hirvonen N., Seitsalo S., Herno A., Kortekangas P. (2007). Surgical or nonoperative treatment for lumbar spinal stenosis?: A randomized controlled trial. Spine.

[4] Fairbank J., Couper J., Davies J., O’brien J. (1980). The Oswestry low back pain disability questionnaire. Physiotherapy.

[5] TEQ Group (1990). EuroQol—A new facility for the measurement of health-related quality of life. Health Policy.

[6] Ghogawala Z., Resnick D.K., Watters W.C., Mummaneni P.V., Dailey A.T., Choudhri T.F., Eck J.C., Sharan A., Groff M.W., Wang J.C. (2014). Guideline update for the performance of fusion procedures for degenerative disease of the lumbar spine. Part 2: Assessment of functional outcome following lumbar fusion. J. Neurosurg. Spine.

[7] Basil G.W., Sprau A.C., Ghogawala Z., Yoon J.W., Wang M.Y. (2020). “Houston, we have a problem”: The difficulty of measuring outcomes in spinal surgery. J. Neurosurg. Spine.

[8] Ahmad H.S., Yang A.I., Basil G.W., Welch W.C., Wang M.Y., Yoon J.W. (2022). Towards personalized and value-based spine care: Objective patient monitoring with smartphone activity data. J. Spine Surg..

[9] Mobbs R.J., Phan K., Maharaj M., Rao P.J. (2016). Physical activity measured with accelerometer and self-rated disability in lumbar spine surgery: A prospective study. Glob. Spine J..

[10] Stienen M.N., Rezaii P.G., Ho A.L., Veeravagu A., Zygourakis C.C., Tomkins-Lane C., Park J., Ratliff J.K., Desai A.M. (2020). Objective activity tracking in spine surgery: A prospective feasibility study with a low-cost consumer grade wearable accelerometer. Sci. Rep..

[11] Ahmad H.S., Yang A.I., Basil G.W., Joshi D., Wang M.Y., Welch W.C., Yoon J.W. (2022). Developing a prediction model for identification of distinct perioperative clinical stages in spine surgery with smartphone-based mobility data. Neurosurgery.

[12] Ahmad H.S., Singh S., Jiao K., Basil G.W., Yang A.I., Wang M.Y., Welch W.C., Yoon J.W. (2022). Data-driven phenotyping of preoperative functional decline patterns in patients undergoing lumbar decompression and lumbar fusion using smartphone accelerometry. Neurosurg. Focus.

[13] Ongsulee P., Chotchaung V., Bamrungsi E., Rodcheewit T. Big data, predictive analytics and machine learning. Proceedings of the 2018 16th International Conference on ICT and Knowledge Engineering (ICT&KE).

[14] Mackenzie A. (2015). The production of prediction: What does machine learning want?. Eur. J. Cult. Stud..

[15] Collins G.S., Reitsma J.B., Altman D.G., Moons K.G. (2015). Transparent reporting of a multivariable prediction model for individual prognosis or diagnosis (TRIPOD): The TRIPOD statement. Ann. Intern. Med..

[16] Von Elm E., Altman D.G., Egger M., Pocock S.J., Gøtzsche P.C., Vandenbroucke J.P., STROBE Initiative (2014). The Strengthening the Reporting of Observational Studies in Epidemiology (STROBE) Statement: Guidelines for reporting observational studies. Int. J. Surg..

[17] Vetter T.R., Schober P. (2018). Regression: The apple does not fall far from the tree. Anesth. Analg..

[18] Chen T., Guestrin C. Xgboost: A scalable tree boosting system. Proceedings of the KDD ‘16: Proceedings of the 22nd ACM SIGKDD International Conference on Knowledge Discovery and Data Mining.

[19] Fawagreh K., Gaber M.M., Elyan E. (2014). Random forests: From early developments to recent advancements. Syst. Sci. Control Eng. Open Access J..

[20] Lee C.K., Hansen H.T., Weiss A.B. (1978). Developmental lumbar spinal stenosis. Pathology and surgical treatment. Spine.

[21] Deme P., Perera A., Chilakapati S., Stutzman S., Singh R.B., Eldridge C.M.B., Caruso J.B., Vira S., Aoun S.G., Makris U.E.M. (2022). Patient and Spine Surgeon Perceptions on Shared Decision-Making in the Treatment of Older Adults Undergoing Corrective Surgery for Adult Spinal Deformity. Spine.

[22] Apple Inc. (2020). Measuring Walking Quality through iPhone Mobility Metrics.

[23] Chang M., Canseco J.A., Nicholson K.J., Patel N., Vaccaro A.R. (2020). The role of machine learning in spine surgery: The future is now. Front. Surg..

[24] Lopez C.D., Boddapati V., Lombardi J.M., Lee N.J., Mathew J., Danford N.C., Iyer R.R., Dyrszka M.D., Sardar Z.M., Lenke L.G. (2022). Artificial learning and machine learning applications in spine surgery: A systematic review. Glob. Spine J..

[25] DelSole E.M., Keck W.L., Patel A.A. (2022). The State of Machine Learning in Spine Surgery: A Systematic Review. Clin. Spine Surg..

[26] Smiti A. (2020). When machine learning meets medical world: Current status and future challenges. Comput. Sci. Rev..

